# A Joint Framework of IMM-LSTM-C Tracking and IBPDO-Based Node Selection for Energy-Efficient Cooperative Tracking in Underwater Acoustic Sensor Networks

**DOI:** 10.3390/s26072277

**Published:** 2026-04-07

**Authors:** Wenbo Zhang, Yadi Hou, Hongbo Zhu

**Affiliations:** School of Information Science and Engineering, Shenyang Ligong University, Shenyang 110159, China; houyadi@yeah.net (Y.H.); hombochu@sina.com (H.Z.)

**Keywords:** underwater acoustic sensor network, cooperative tracking, node selection, IBPDO, sensor scheduling, energy efficiency

## Abstract

The increasing deployment of underwater vehicles demands accurate and energy-efficient target tracking in sensor networks. However, existing approaches have largely addressed tracking accuracy and energy efficiency in isolation, and a system-level framework that jointly optimizes both remains lacking. To address this gap, this paper proposes a joint optimization framework with two main contributions. First, to improve tracking accuracy under complex maneuvering conditions, we develop an Interactive Multi-Model using Long Short-Term Memory Classification (IMM-LSTM-C) algorithm, which integrates multi-step model likelihoods into an LSTM network for precise motion classification, achieving a 7.1% accuracy improvement over IMM-BP. Second, to reduce network energy consumption while maintaining tracking performance, we introduce an Improved Binary Prairie Dog Optimization (IBPDO) algorithm for node selection, enhanced with Cauchy mutation and opposition-based learning. Simulation results show that IBPDO achieves 6.1–8.2% higher accuracy than BWOA and reduces energy consumption by 12% compared to LNS. Furthermore, the complete joint framework demonstrates synergistic effects, reducing tracking error by 19.3% and energy consumption by 15.4% over the IMM + LNS baseline. The proposed framework provides an effective balance between tracking accuracy and energy efficiency in underwater acoustic sensor networks.

## 1. Introduction

Underwater target tracking is a fundamental capability for diverse applications, including maritime security, marine environmental monitoring, and autonomous underwater vehicle (AUV) navigation. Traditional approaches relying on ship-towed or submarine-mounted sonar arrays suffer from high operational costs, limited coverage, and strong dependence on host platform mobility, making them unsuitable for long-term, large-scale surveillance [1]. In contrast, underwater acoustic sensor networks (UASNs) have emerged as a promising alternative due to their self-organizing architecture, low observability, and potential for persistent, wide-area monitoring [2].

However, the practical deployment of UASNs is severely constrained by the limited battery capacity of underwater nodes. Despite significant efforts to improve either tracking accuracy [3,4,5,6] or energy efficiency [7,8,9,10,11] in isolation, a system-level framework that jointly optimizes both remains lacking. Conventional energy harvesting methods—such as solar or wind—are ineffective in deep or turbid waters [12], rendering continuous activation of all sensors energetically unsustainable. Consequently, intelligent sensor management strategies that dynamically select a minimal subset of cooperative nodes are essential to balance tracking accuracy with energy conservation [13,14].

At the physical layer, recent advances have demonstrated potential for energy-efficient underwater communications. For instance, Cai et al. proposed a joint energy and correlation detection assisted non-coherent OFDM-DCSK system [15] and an OFDM-based differential cyclic-shifted DCSK system [16] that reduce energy consumption by activating only a subset of subcarriers. These physical-layer techniques are complementary to network-layer node selection strategies, as the energy savings from efficient modulation can be further enhanced by intelligent scheduling algorithms. However, at the network layer—the focus of this work—two critical challenges remain unaddressed by existing literature [3,4,5,6,7,8,9,10,11].

First, most tracking filters [3,4,5,6] rely on short-term information (e.g., single-step likelihoods), which makes them vulnerable to measurement noise and leads to inaccurate motion model identification during target maneuvers. Second, existing node selection algorithms [7,8,9,10,11] treat sensor scheduling as a static optimization problem and often suffer from premature convergence in high-dimensional binary spaces. Critically, these two modules are typically designed in a decoupled manner [3,4,5,6,7,8,9,10,11], missing the opportunity for joint optimization where node selection is informed by tracking confidence and the tracker adapts to the selected nodes.

To address these interconnected gaps, this paper makes two main contributions:

1. To overcome the short-term dependency limitation of conventional tracking filters [8,9,10,11], we propose an Interactive Multi-Model using Long Short-Term Memory Classification (IMM-LSTM-C) algorithm. It integrates Long Short-Term Memory (LSTM) networks with the Interacting Multiple Model (IMM) framework to capture long-term temporal dependencies in motion model likelihoods, thereby achieving more accurate and stable model probability estimates.
2. To address the limitations of existing binary node selection algorithms [7,8,9,10,11] and the decoupled design paradigm [3,4,5,6,7,8,9,10,11], we introduce an Improved Binary Prairie Dog Optimization (IBPDO) algorithm for node selection. Enhanced with Cauchy mutation and hybrid opposition-based learning, and adopting a V-shaped transfer function for binary conversion, the IBPDO algorithm formulates node selection as a multi-objective optimization problem with a weighted sum of tracking accuracy and network energy consumption. This enables dynamic scheduling of node subsets that optimally balance the accuracy–energy trade-off.

The remainder of this paper is structured in the following manner. Section 2 provides a comprehensive literature review. Section 3 details the proposed IMM-LSTM-C tracking algorithm. Section 4 presents the IBPDO-based node selection framework. Section 5 concludes the paper.

## 2. Related Work

### 2.1. Tracking Filtering Methods

The core technical challenge in UASN-based target tracking lies in the inherent nonlinear relationship between acoustic measurements and target states. Addressing the spatially varying characteristics of underwater sound speed, Liu et al. [3] proposed a double-layer weighted unscented Kalman filter algorithm that adaptively weights measurement noise by incorporating sound speed profile information, effectively enhancing underwater tracking accuracy.

To address target maneuverability, multiple model estimation methods have been widely adopted. The IMM algorithm proposed by Blom and Bar-Shalom [4] achieves adaptive state estimation in dynamic environments by fusing multiple candidate motion models. Building upon this foundation, Lee and Park [5] further introduced a situation-aware adaptive transition probability matrix update method that dynamically adjusts model probabilities through real-time assessment of model matching, significantly improving tracking performance in complex maneuvering scenarios. Wang et al. [6] introduced deep learning into passive sonar target tracking, utilizing neural networks to learn target motion patterns and providing a new technical pathway for traditional filtering methods.

Despite these advances, the tracking filtering methods discussed above [3,4,5,6] share a common limitation: they all update motion model probabilities based solely on current and immediately preceding observations. Liu et al. [3] focus on measurement noise adaptation, Lee and Park [5] adjust transition probabilities, and Wang et al. [6] use neural networks for pattern learning. However, none of these approaches exploit long-term temporal dependencies in multi-step likelihood sequences to stabilize model probability estimates under persistent high noise. This limitation, which persists across existing IMM variants, motivates our proposed IMM-LSTM-C algorithm described in Section 3.

### 2.2. Node Selection and Scheduling Strategies

In recent years, swarm intelligence (SI) algorithms have gained increasing attention for node selection and scheduling in wireless sensor networks due to their ability to handle complex combinatorial optimization problems. Algorithms such as Particle Swarm Optimization (PSO) [7], Genetic Algorithm (GA) [8], Whale Optimization Algorithm (WOA) [9], and Salp Swarm Algorithm (SSA) [10] have been adapted to binary versions for sensor activation problems.

However, when applied to UASN node selection, these algorithms exhibit certain limitations that are particularly detrimental in the context of cooperative target tracking.

First, PSO and GA are prone to premature convergence in high-dimensional binary spaces. In our node selection problem, the candidate set consists of the 20 nearest sensors, from which 3–5 nodes must be selected. This yields a combinatorial search space of size $\left(\begin{array}{c} 20\\ 5 \end{array}\right)=15,504$ possible combinations at each time step. Premature convergence in such a space often leads the algorithm to settle on suboptimal node subsets that either provide poor spatial diversity (degrading tracking accuracy) or activate redundant nodes (wasting energy).

Second, BWOA, despite its fast convergence, suffers from insufficient exploration capability. In underwater environments, sensor measurements are subject to high acoustic noise (standard deviation σ = 60 m in our setup). The fitness landscape of node selection is therefore highly irregular, with many locally optimal subsets that do not generalize well to the actual target trajectory. BWOA’s tendency to converge quickly to the first promising region makes it prone to selecting node subsets that are optimal for a particular noise realization but suboptimal for long-term tracking.

Third, BSSA features simple implementation but lacks effective exploitation mechanisms. In cooperative tracking, node selection must be performed at each time step (every 10 s in our simulation). BSSA’s slow convergence in later optimization stages means it may not reach a high-quality solution within the available time budget, leading to tracking delays or outdated node selections.

These limitations motivate the proposed IBPDO algorithm, which is specifically designed to balance exploration and exploitation in the binary, high-dimensional, and noise-affected node selection problem of UASN target tracking.

### 2.3. Research Gap in Joint Optimization Methods

Synthesis of the existing literature reveals a critical limitation: node selection and state estimation are predominantly addressed within a decoupled design paradigm. Research on tracking filtering typically assumes continuous activation of all nodes, while studies on node selection treat the filtering method as a static back-end module. This fragmented approach results in node combinations that are suboptimal for the prevailing target dynamics, ultimately preventing the system from achieving a Pareto-optimal balance between estimation accuracy and energy consumption.

Qin et al. [11] attempted to bridge this gap by proposing a method combining node scheduling with unscented Kalman filter (UKF) for non-cooperative target tracking scenarios. However, their approach still lacks substantive bidirectional interaction between node selection and state estimation modules. Furthermore, existing scheduling algorithms predominantly employ continuous relaxation or standard heuristic methods when addressing the discrete combinatorial optimization problem of node activation, lacking efficient global optimization mechanisms specifically designed for binary search spaces. This limitation is particularly pronounced in energy-constrained UASN deployment environments with limited node resources.

### 2.4. Research Positioning and Contributions of This Paper

From the literature reviewed above, three interconnected research gaps emerge. First, despite the advances in tracking filtering methods, they all share an unresolved limitation: they update motion model probabilities based only on current and immediately preceding observations (i.e., short-term temporal context). None of these approaches exploit long-term temporal dependencies in multi-step likelihood sequences to stabilize model probability estimates under high measurement noise. LSTM-enhanced methods for integrating such long-term likelihood sequences remain largely unexplored in underwater contexts. Second, existing node selection methods typically optimize for either tracking accuracy or energy consumption, but rarely both within a unified framework, and the optimization algorithms employed are often ill-suited for the discrete, combinatorial nature of node activation in UASNs. Third and most fundamentally, the lack of genuinely joint optimization frameworks prevents closed-loop adaptation where selection decisions are informed by tracking performance and tracking algorithms account for the properties of selected sensors, limiting the system’s ability to achieve optimal accuracy–energy trade-offs over time.

## 3. LSTM-Based Target Tracking Algorithm for Motion Model Classification

Underwater moving targets often exhibit flexible and diverse motion characteristics, making them unsuitable for description using a single motion model. Consequently, the IMM is commonly employed to characterize their motion. However, the conventional IMM algorithm relies solely on the estimation results of each motion model from the previous time step and the observations at the current time step. This limitation leads to insufficient model discriminability and significant fluctuations in model probabilities, resulting in degraded target tracking performance.

To address this issue, the IMM algorithm can be integrated with LSTM networks, giving rise to the proposed IMM-LSTM-C. LSTM networks excel at capturing long-term dependencies in time series data, enabling the synthesis of information acquired by sensors over an extended period to achieve more accurate motion model identification. This paper proposes a target tracking algorithm that leverages LSTM networks for motion model classification, wherein the likelihood function values of the IMM over multiple time steps are input into the LSTM network to determine the target’s motion model.

**Figure 1:**
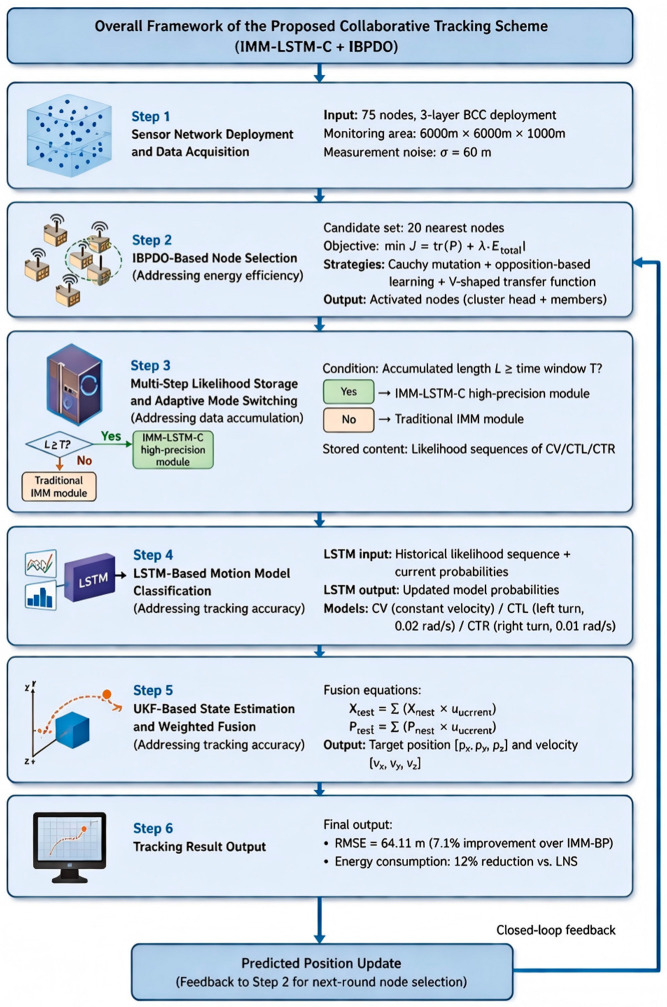
Overall framework and workflow of the proposed collaborative tracking scheme.

Figure 1 presents the overall framework of our proposed collaborative tracking scheme, which we refer to as IMM-LSTM-C + IBPDO. A three-layer underwater acoustic sensor network consisting of 75 nodes is deployed within the monitoring region to collect noisy target observations. Based on this setup, an IBPDO-based node selection strategy is introduced to pick out the optimal subset of activated nodes from the candidate neighboring nodes, striking a better balance between tracking performance and energy consumption. A multi-step likelihood storage mechanism is then constructed, with the processing mode adaptively switched depending on whether the accumulated sequence length has reached a predefined time window. When sufficient historical information is available, the high-precision IMM-LSTM-C module is activated; otherwise, the traditional IMM module takes over to maintain tracking continuity. The stored likelihood sequences, together with the current model probabilities, are fed into an LSTM-based motion model classification module, which dynamically updates the mode probabilities for different target motion patterns. With these updated probabilities, the UKF-based state estimation and weighted fusion module further integrates the state estimates obtained under multiple motion models, yielding the final estimates of target position and velocity. Tracking results, including accuracy and energy-efficiency metrics, are then produced. Meanwhile, the predicted target position is fed back to the node selection stage, forming a closed-loop optimization framework for the next round of collaborative tracking.

### 3.1. Target Tracking Pipeline Based on LSTM Motion Model Classification

The overall procedure of target tracking using LSTM for motion model classification is described below.

Upon target detection, an underwater acoustic sensor node initiates an acoustic signal transmission toward the detected target. The relative distance between the node and the target is calculated from the time delay between the transmitted acoustic wave and the received echo signal.

Initially, the node employs the conventional IMM algorithm to produce a coarse estimate of the target’s motion direction. It then forwards the relevant data—including motion model likelihoods, state estimates for each individual model, and the corresponding covariance estimates—to the neighboring sensor node situated along the estimated direction. This procedure is executed iteratively until the node accumulates sufficient historical data, namely the motion model likelihood values over multiple consecutive time steps up to the current time.

Algorithm 1 illustrates the data accumulation and adaptive tracking mode switching process for sensor nodes. Each node collects distance measurements and external information from neighboring nodes. When the accumulated data length reaches the predefined time window, the LSTM-based tracking module is activated; otherwise, the traditional IMM module is used to maintain tracking continuity. The resulting state estimate is then forwarded to nodes in the predicted target direction. The specific steps are as described below.
**Algorithm 1.** Process of Data Accumulation in Sensor Nodes**Input**: Distance measurements from the sensor to the target; Target information from other nodes**Output**: Target position and velocity estimation X_est; State estimation covariance P_est; Current motion model probability u_current1: Measure the distance from the target to the sensor node2: **if** other nodes sent likelihood function and related information **then**3:          Receive information from other nodes4:          **if** length of accumulated information ≥ time window length **then**5:                X_est, P_est, u_current ← LSTM-based motion model classification for target tracking6:          **else**7:                X_est, P_est, u_current ← Traditional IMM target tracking8:          **end if**9: **end if**10: Send target information to other nodes in the target’s moving direction11: **return** X_est, P_est, u_current

Once sufficient data have been accumulated, the sequence of motion model likelihood values within the sliding window is fed into an LSTM neural network. The LSTM outputs the probability distribution over the target’s motion models at the current time step. These probabilities are subsequently used by the IMM algorithm to fuse the state estimates from the individual models, yielding the final estimates of the target’s position and velocity.

Algorithm 2 presents the target tracking process LSTM-based motion model classification. Given the current distance measurement, state estimates from each motion model, and the historical likelihood matrix, the algorithm first computes the current likelihood values for each model. These values are then combined with the historical likelihood matrix and fed into the LSTM network to obtain refined model probabilities, which are subsequently used to fuse the state estimates from all models. The detailed process is described below.
**Algorithm 2.** Target Tracking Process for Motion Model Classification Using Long Short-Term Memory**Input:** Distance measurement from the sensor to the target (dist_mea); State estimates of each model (Xn_est); Covariance estimates of each model (Pn_est); Motion model likelihood value matrix over multiple time steps (u_history)**Output:** Estimated target position and velocity (X_es*t*); Covariance of target state estimation (P_est); Current target motion model probability (u_current)1: **for** i = 1 to 3 **do**2:          u_current(i) ← move_model_likelihood_func (dist_mea, Xn_est(i), Pn_est(i))3: **end for**4: u_current ← softmax (u_current)5: u_current ← LSTMNet (u_history, u_current)6: **for** i = 1 to 3 **do**7:          X_est ← X_est + Xn_est(i) ∗ u_current(i)8:          P_est ← P_est + Pn_est(i) ∗ u_current(i)9: **end for**10: **return** X_est, P_est, u_current

### 3.2. Simulation Experiment and Analysis

Following the design of the neural network architecture, training requires a dataset. Given the practical difficulty and cost associated with acquiring real observational data of underwater target trajectories, a simulation-based dataset is generated using MATLAB for model training [17].

**Figure 2:**
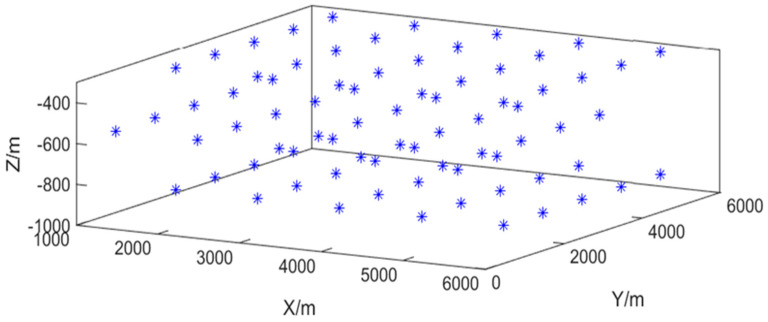
Schematic diagram of sensor position arrangement.

As depicted in Figure 2, the simulation is set in a three-dimensional space of dimensions 6000 m × 6000 m × 1000 m [18]. Within this environment, the target motion is governed by three fundamental kinematic models: Constant Velocity (CV), Coordinated Turn Left (CTL), and Coordinated Turn Right (CTR). The initial state vector is defined as [500, 5, 1000, 10, −900, 1] (units: meters for position and meters per second for velocity, assuming the standard order [px, vx, py, vy, pz, vz]).

The target’s motion sequence is described below. It maintains CV motion for 20 time steps, performs a CTL maneuver with an angular velocity of 0.02 rad/s for 10 time steps, executes a CTR maneuver at 0.01 rad/s for 20 time steps, and concludes with a final CV phase lasting 30 time steps [19]. In the simulation scenario, the system noise for the CV, CTL, and CTR motion models is modeled as additive white Gaussian noise (AWGN) with a standard deviation of 0.02 (in applicable state units).

A total of 75 sensors are deployed to monitor the target. They are distributed across three horizontal layers, with 25 sensors per layer. Within each layer, sensors are arranged in a uniform 5 × 5 grid, with an inter-sensor spacing of 1000 m along both the X and Y axes. To achieve a spatially diverse three-dimensional array, the middle layer is offset by −500 m along both the X and Y axes relative to the outer layers, forming a configuration analogous to a body-centered cubic (BCC) lattice [20]. In the vertical (*Z*-axis) direction, the three layers are positioned at depths of 300 m, 600 m, and 900 m, respectively. The complete parameter settings are detailed in Table 1.

Let the three-dimensional position of sensor *i* be denoted as *pi* = (*xi*, *yi*, *zi*). The sensor measures the range (distance) to the target. Thus, the measurement model can be expressed as

(1) $$Z_{k}^{i}=h_{k}^{i}\left(X_{k}\right)+{\;v}_{k}^{i}$$

where

$Z_{k}^{i}$: measurement from sensor *i* at time step *k*.

$X_{k}$: target state vector at time *k*, including position and velocity.

$h_{k}^{i}\left(\cdot \right)$: nonlinear measurement function that maps the target state to the expected range measurement.

$v_{k}^{i}$: measurement noise, assumed to be zero-mean Gaussian white noise with standard deviation *σ* = 60, and covariance matrix R.

The measurement function is specifically defined as the Euclidean distance between the target position and the sensor position.

(2) $$h_{k}^{i}\left(X_{k}\right)=\sqrt{(x_{k}-x_{i}{)}^{2}+(y_{k}-y_{i}{)}^{2}+(z_{k}-z_{i}{)}^{2}}$$

where

$\left(x_{k},y_{k},z_{k}\right)$: predicted position of the target at time *k*.

$\left(x_{i},y_{i},z_{i}\right)$: fixed three-dimensional position of sensor *i*.

**Figure 3:**
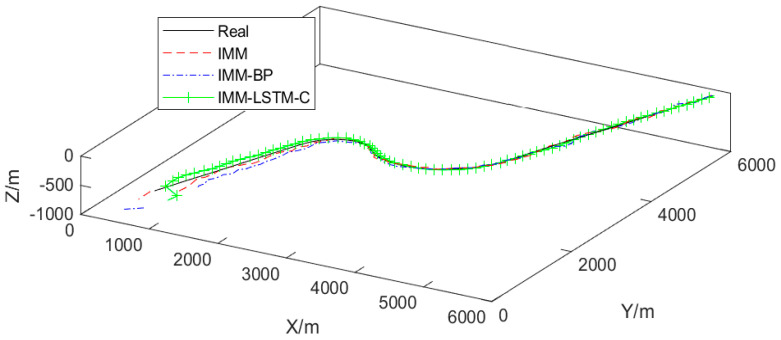
Each algorithm tracks the trajectory of a moving target.

The simulations and dataset generation were performed in MATLAB R2023a on a computing platform equipped with an Intel Core i5-10400F CPU. The target tracking trajectories of each algorithm are shown in Figure 3. A total of 30,000 independent trajectories were generated for the experiment. Each trajectory comprises 80 discrete time steps with a sampling interval of 10 s between consecutive steps.

The generated dataset was randomly split into training, validation, and test sets, accounting for 70%, 15%, and 15% of the total data, respectively. As the motion model labels (CV, CTL, CTR) are categorical without inherent ordinal relationships, they were converted into a one-hot encoded format. In this representation, the true model at a given time step is encoded as 1, while the other two models are encoded as 0. Specifically, the encoding vectors are CV → [1, 0, 0], CTL → [0, 1, 0], and CTR → [0, 0, 1].

**Figure 4:**
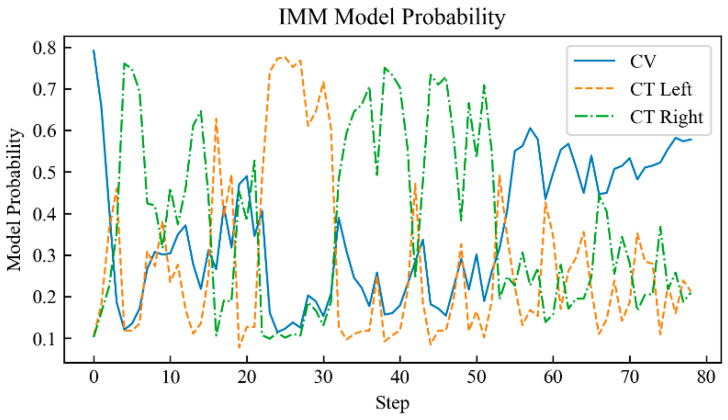
Probability curve of the target motion model judged by the IMM algorithm.

**Figure 5:**
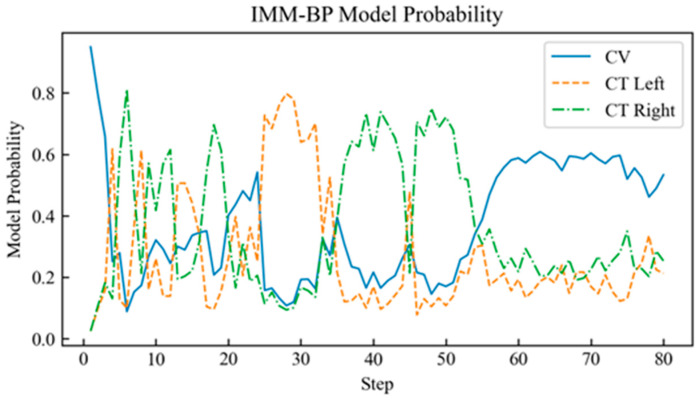
Probability curve of the target motion model judged by the IMM-BP algorithm.

**Figure 6:**
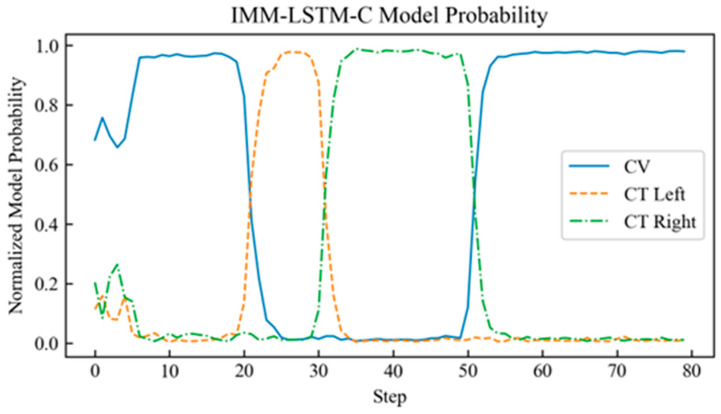
Probability curve of the target motion model judged by the IMM-LSTM-C algorithm.

The proposed neural network component of the IMM-LSTM-C algorithm was implemented using PyTorch 2.0.1 in a Python 3.10.10 environment, with the maximum number of training epochs set to 3000. To benchmark its performance, comparative experiments were carried out against two baseline algorithms: the standard IMM algorithm and an IMM variant that integrates Kalman filter intermediate variables with a standard backpropagation-trained neural network (referred to as IMM-BP). The probability curves of the target motion models judged by each algorithm are shown in Figure 4, Figure 5 and Figure 6.

Figure 4, Figure 5 and Figure 6 show the motion model probability output curves of the IMM algorithm, the IMM-BP algorithm, and the proposed IMM-LSTM-C algorithm, respectively, during target maneuvering. In each figure, the horizontal axis represents the simulation time step, and the vertical axis represents the model probability. The three curves correspond to the CV, CTL, and CTR motion models, respectively. As shown in Figure 4, the model probability curves of the traditional IMM algorithm fluctuate significantly and cannot stably distinguish between the CTL and CTR models during turning maneuvers. In Figure 5, although the IMM-BP algorithm shows some improvement, its probability outputs still exhibit considerable uncertainty. In contrast, as shown in Figure 6, the model probability curves of the IMM-LSTM-C algorithm are much smoother and can quickly and accurately converge to the correct motion model near the switching points, significantly improving the recognition capability of target maneuvering modes.

The tracking accuracy of each algorithm is further investigated through 100 independent Monte Carlo experiments. The tracking accuracy results of different algorithms are presented in Table 2.

It can be seen that in this scenario, the target maneuvers flexibly with two turns. Compared with the IMM algorithm, the tracking error of the IMM-LSTM-C algorithm is reduced by 11.2% and by 7.1% compared with the IMM-BP algorithm.

The IMM-LSTM-C algorithm integrates historical likelihood sequences over multiple time steps via an LSTM network, enabling more accurate motion model identification. However, both the IMM and IMM-BP algorithms only utilize the estimation and measurement information of the previous and current moments, and cannot integrate information over a relatively long period. Consequently, they fail to accurately identify the target motion model, resulting in inferior tracking performance.

## 4. Node Cooperative Tracking Algorithm

### 4.1. Optimization Analysis of Cooperative Tracking

Sensor node selection and scheduling for target tracking operate in a closed-loop manner. At each time step, sensor measurements are used to estimate the target state. This estimate is then fed back into the selection and scheduling module to activate an optimal subset of sensors for acquiring measurements at the next time step. The process iterates until tracking concludes.

To implement this framework, appropriate performance metrics must first be defined based on practical constraints, in order to quantify the tracking capability of underwater acoustic sensor nodes with respect to the target. The objective function also incorporates additional factors such as energy consumption, and an optimization algorithm is employed to solve the resulting problem. This enables the dynamic selection of one or more sensors to guarantee a required tracking accuracy while avoiding excessive energy consumption [21].

To minimize network energy usage, a minimal number of active sensor nodes should be selected for tracking. Conversely, to achieve high tracking accuracy, the trace of the posterior estimation error covariance matrix $P_{t|t}$, denoted as $tr(P_{t|t})$, is included as the primary accuracy-related term in the objective function [22].

In UASNs, node energy consumption is primarily attributed to three functional modules: sensing, computation, and communication. Among these, the communication module accounts for the largest portion of the total energy consumption during active operation. The energy consumption of the communication module arises from four operational states: transmission, reception, idle, and sleep. Transmission consumes the most energy, followed by reception and idle states, while the sleep state incurs the lowest energy cost.

In cooperative tracking, sensor nodes must transmit aggregated target information to the fusion center via the acoustic channel, making target tracking an energy-demanding application in UASNs [23].

Assume that the minimum power required to receive one bit of data successfully is $P_{r}$. Then, the energy consumed by node A to transmit n bits to node B over a distance of d meters in a shallow water environment is given by

(3) $$E_{tx}=nP_{r}A(d)$$

where

$E_{tx}$: energy consumed for transmission (Joules).

*n*: number of bits transmitted.

$P_{r}$: minimum power required to successfully receive one bit of data (J/bit).

A(d): amplification coefficient accounting for underwater noise and signal absorption.

(4) $$A\left(d\right)={\left(\frac{d}{1000}\right)}^{m}\alpha^{\frac{d}{1000}}$$

where

d: transmission distance (meters).

m: energy spreading factor (*m* = 1 for cylindrical spreading, *m* = 2 for spherical spreading).

α: absorption coefficient, which is frequency-dependent.

(5) $$\alpha =10^{\frac{a}{10}}$$

where

a: absorption loss in dB/km, defined by Thorp’s formula.

(6) $$a=\frac{0.11f^{2}}{1+{\;f}^{2}}+\frac{44f^{2}}{4100+f^{2}}+2.75\times 10^{-4}f^{2}+0.003$$

where

f: frequency of the acoustic signal (kHz).

The total energy consumption of all selected nodes within one time step is given by

(7) $$E_{total}=\sum_{i=1}^{N_{s}}E_{tx}^{i}$$

where

$E_{total}$: total energy consumption of the selected sensor nodes in one time step.

$N_{s}$: number of sensor nodes selected to participate in tracking.

$E_{tx}^{i}$: transmission energy consumption of the i-th selected node.

In the simulation experiment, the trace of the posterior estimation covariance matrix, $tr\left(P_{t|t}\right)$, is on the order of several thousand. To balance the energy consumption term with the tracking accuracy term in the objective function, the energy consumption is scaled by a coefficient λ=50.

In the node scheduling cooperative tracking problem, the objective function is

(8) $$F=tr\left(P_{t|t}\right)+\lambda E_{total}$$

where

F: objective function value to be minimized.

$tr\left(P_{t|t}\right)$: trace of the posterior estimation error covariance matrix, representing tracking accuracy.

λ: balancing coefficient (set to 50 in simulations) to scale the energy term to a comparable order of magnitude as the accuracy term.

$E_{total}$: total energy consumption, as defined in Equation (7).

### 4.2. Node Selection Strategy Based on Local Information

A simple underwater node selection strategy is the Local Node Selection (LNS) strategy based on local information. The covariance of the target state estimate at time k+1 obtained at time k is used as the covariance σ of the one-step prediction of the target state. According to the predicted value of the target state and the covariance of the one-step prediction, a sphere centered at the predicted target position with a radius of 3σ is plotted, and the one-step prediction result is broadcast to neighboring nodes. The cluster head adjusts its broadcast communication distance $R_{comm}$ to $min\left\{\;R_{max},r_{s}+\;3\sigma +\;l\;\right\}$, where $R_{max}$ denotes the maximum communication distance of the cluster head node, l is the distance from the cluster head to the predicted target position at time k + 1, and r_s represents the sensing radius of the node. After receiving the information sent by the cluster head, neighboring nodes calculate their own distance $d_{i}$ to the predicted target position as follows:

(9) $$d_{i}=\sqrt{(x_{i}-\hat{x}{)}^{2}\;+\;(y_{i}-\hat{y}{)}^{2}\;+\;(z_{i}-\hat{z}{)}^{2}}$$

where

$d_{i}$: distance from node *i* to the predicted target position.

($x_{i},y_{i},z_{i}$): coordinates of node *i*.

($\hat{x},\hat{y},\hat{z}$): predicted target position at the next time step.

If the sensing range of a node overlaps with the predicted 3σ sphere, i.e., $d_{i}\leq r\;+\;3\sigma$, the node will be woken up from the sleep mode. To fuse the data from multiple sensors, a new cluster head needs to be selected from the woken nodes. Herein, the node closest to the predicted target position is selected as the new cluster head, and the remaining nodes act as cluster member nodes. After the sensors acquire the measurement information of the moving target and transmit the information to the fusion center, the fusion center aggregates the measurement results from all sensors to derive a more accurate estimate of the target state. First, the covariance of the fusion estimation error is calculated.

(10) $$P_{k+1}={\left[\sum_{i=1}^{N}{\left(P_{k+1}^{i}\right)}^{-1}\;-\left(N-1\right){\left(P_{k+1|k}\right)}^{-1}\right]}^{-1}$$

where

$P_{k+1}$: fused estimation error covariance at time *k* + 1.

N: number of nodes participating in tracking.

$P_{k+1}^{i}$: estimation error covariance of node *i* at time *k* + 1, computed by its local filter.

$P_{k+1|k}$: prediction covariance of the target state at time *k* + 1 given information up to time *k*.

Both of these two terms are derived from the filtering algorithm. The fused estimate of the target state is calculated after acquiring the local estimates of each node, which rely on the measurement information:

(11) $${\hat{X}}_{k+1}=P_{k+1}\left[\sum_{i=1}^{N}{P_{k+1}^{i}}^{-1}{\hat{X}}_{k+1}^{i}\;-\;\left(N\;-\;1\right)P_{k+1|k}^{-1}{\hat{X}}_{k+1|k}\right]$$

where

${\hat{X}}_{k+1}$: fused target state estimate at time *k* + 1.

${\hat{X}}_{k+1}^{i}$: local state estimate of node *i* at time *k* + 1.

${\hat{X}}_{k+1|k}$: predicted target state at time *k* + 1 based on time *k*.

After three or more sensors have measured the distance to the target, the least squares solution of the system of equations can be obtained via the trilateration method to solve for the target position. Trilateration requires simultaneous measurements from sensors, yet each sensor may have a different measurement period. Considering the potential delays in underwater acoustic channels, directly inputting the measurement information into the Unscented Kalman Filter also enables accurate target tracking.

### 4.3. Prairie Dog Optimization Algorithm

The PDO algorithm is a swarm intelligence algorithm proposed by Ezugwu et al. [24] in 2022. Prairie dogs are herbivorous burrowing rodents primarily found in the Great Plains and southwestern deserts of the United States, as well as in the plains and plateaus of Canada and Mexico. Each prairie dog habitat houses 15–26 family units, known as coteries. A coterie typically consists of one adult male, two to three adult females, and their offspring.

The algorithm employs four types of prairie dog-inspired behaviors to address the two fundamental phases of optimization: exploration and exploitation [25]. Foraging and burrowing behaviors underpin the exploration phase. Specifically, prairie dogs excavate burrows near abundant food sources; when a source is depleted, they seek new ones and establish new burrows nearby, thereby exploring the entire habitat—interpreted as the problem space—to locate new candidate solutions. Additionally, prairie dogs emit two distinct types of vocalizations: one signaling predator presence and the other indicating food availability.

The prairie dog optimization algorithm is based on the following assumptions:

1. A prairie dog habitat contains m family coteries, with each coterie consisting of n prairie dogs. Each prairie dog belongs to one coterie.
2. The habitat, which corresponds to the problem’s search space, is divided into multiple regions. Each family inhabits one region, and each region contains 10 to 100 burrow entrances.
3. Prairie dogs emit two distinct acoustic signals: an alarm call indicating the presence of a predator, and a food call signaling the discovery of a new food source. They construct new burrows near new food sources.
4. Foraging and burrowing activities (exploration), as well as communication and anti-predator behaviors (exploitation), involve only members of the same family.
5. All coteries in the habitat perform these activities simultaneously. The exploration and exploitation phases are repeated for m iterations.

Like other population-based algorithms, the prairie dog optimization algorithm randomly initializes the positions of the prairie dogs. The population of prairie dogs acts as a group of search agents, with the position of each individual represented by a vector in a d-dimensional space.

#### 4.3.1. Initialization

The position of a prairie dog within a given family coterie can be represented by a vector. The positions of all coteries (CTs) in a habitat are defined as follows:

(12) $$CT=\left[\begin{array}{ccccc} {CT}_{1,1} & {CT}_{1,2} & \cdots & {CT}_{1,d-1} & {CT}_{1,d}\\ {CT}_{2,1} & {CT}_{2,2} & \cdots & {CT}_{2,d-1} & {CT}_{2,d}\\ \vdots & \vdots & {CT}_{i,j} & \vdots & \vdots \\ {CT}_{m,1} & {CT}_{m,2} & \cdots & {CT}_{m,d-1} & {CT}_{m,d} \end{array}\right]$$

where

CT: matrix representing the positions of all coteries in the habitat.

*m*: number of coteries (family groups).

*d:* dimension of the optimization problem.

${CT}_{i,j}$: value of the *j*-th dimension for the *i*-th coterie.

The positions of all prairie dogs within a coterie are given by

(13) $$PD=\left[\begin{array}{ccccc} PD_{1,1} & PD_{1,2} & \cdots & PD_{1,d-1} & PD_{1,d}\\ PD_{2,1} & PD_{2,2} & \cdots & PD_{2,d-1} & PD_{2,d}\\ \vdots & \vdots & PD_{i,j} & \vdots & \vdots \\ PD_{n,1} & PD_{n,2} & \cdots & PD_{n,d-1} & PD_{n,d} \end{array}\right]$$

where

PD: matrix representing the positions of all prairie dogs within a single coterie.

*n*: number of prairie dogs in a coterie.

*d*: dimension of the optimization problem.

$PD_{i,j}$: value of the *j*-th dimension for the *i*-th prairie dog in the coterie.

(14) $${CT}_{i,j}=U\left(0,1\right)\times \left({UB}_{j}-{LB}_{j}\right)+{\;LB}_{j}$$

where

$U\left(0,1\right)$: a uniformly distributed random number between 0 and 1.

${UB}_{j}\;and\;{LB}_{j}$: upper and lower bounds of the j-th dimension of the global optimization problem.

(15) $${PD}_{i,j}=U\left(0,1\right)\times \left({ub}_{j}-{lb}_{j}\right)+{lb}_{j}$$

where

${ub}_{j}=\frac{{UB}_{j}}{m}$ and ${lb}_{j}=\frac{{LB}_{j}}{m}$: upper and lower bounds for the prairie dogs’ search space within their coterie.

The fitness value of each prairie dog’s position is evaluated by inputting the solution vector into the defined objective function:

(16) $$f\left(PD\right)=\left[\begin{array}{ccccc} f_{1}([PD_{1,1} & PD_{1,2} & \cdots & PD_{1,d-1} & PD_{1,d}])\\ f_{2}([PD_{2,1} & PD_{2,2} & \cdots & PD_{2,d-1} & PD_{2,d}])\\ \vdots & \vdots & \cdots & \vdots & \vdots \\ f_{n}([PD_{n,1} & PD_{n,2} & \cdots & PD_{n,d-1} & PD_{n,d}]) \end{array}\right]$$

where

$f\left(PD\right)$: a vector of fitness values for all prairie dogs in a coterie.

$f_{1}(\cdot )$: the objective function evaluating the quality of the *i*-th prairie dog’s position.

The fitness value for each prairie dog reflects the quality of the food source at its location, the suitability of the site for digging a new burrow, and the reliability of the alarm signal regarding predator presence.

The prairie dog optimization algorithm utilizes foraging and burrowing activities to explore the problem space. Prairie dogs excavate burrows around abundant food sources. When a food source is depleted, they search for a new one and construct new burrows around it, thereby exploring the entire habitat (i.e., the problem space) to discover new solutions.

The prairie dog optimization algorithm employs four strategies. The first two strategies are used for exploration when $iter<\frac{Max_{iter}}{4}$ or $\frac{Max_{iter}}{4}\leq iter<\frac{Max_{iter}}{2}$. The latter two strategies are used for exploitation when $\frac{Max_{iter}}{2}\leq iter<3\frac{Max_{iter}}{4}$ or $3\frac{Max_{iter}}{4}\leq iter\leq Max_{iter}$.

#### 4.3.2. Exploration Process

**Figure 7:**
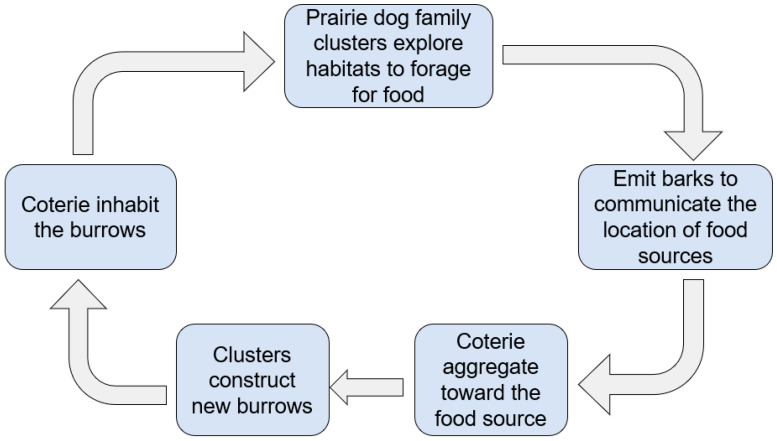
Exploration phase of the prairie dog optimization algorithm.

The exploration process of the prairie dog optimization algorithm is shown in Figure 7. The first strategy involves family members searching for new food sources. This search process can be modeled as a Lévy flight, which is effective for exploring large areas rather than conducting intensive local searches [26]. Once a food source is located, members emit a specific call to inform others of the discovery. The members then select the optimal food source for foraging and proceed to excavate new burrows nearby. During the exploration phase of the algorithm, when $iter<\frac{Max_{iter}}{4}$, the foraging position is updated according to Equation (17):

(17) $${PD}_{i+1,j+1}={GBest}_{i,j}-{\;eCBest}_{i,j}\times \rho -{\;CPD}_{i,j}\times L\overset{’}{e}vy\left(n\right)$$

where

${GBest}_{i,j}$: the global best solution found so far.

${eCBest}_{i,j}$: the influence of the current best solution.

ρ: attractiveness parameter of the food-source discovery call (set to 0.1).

${CPD}_{i,j}$: cumulative effect of all prairie dogs in the coterie.

$L\overset{’}{e}vy\left(n\right)$: a random number generated by a Lévy flight, used for exploring large areas.

(18) $${eCBest}_{i,j}={GBest}_{i,j}\times \Delta +\frac{{PD}_{i,j}\times mean\left({PD}_{n,m}\right)}{{GBest}_{i,j}\times \left({UB}_{j}-{LB}_{j}\right)+\Delta }$$

where

Δ: a small value representing minute differences between individual prairie dogs (set to 0.005).

$mean\left({PD}_{n,m}\right)$: the mean position of all prairie dogs in the coterie.

(19) $${CPD}_{i,j}=\frac{{GBest}_{i,j}-{\;rPD}_{i,j}}{{GBest}_{i,j}+\Delta }$$

where

${rPD}_{i,j}$: the position of a randomly selected prairie dog solution from the population.

The second strategy adjusts the excavation intensity according to the quality of the current food source. The excavation intensity decreases as the number of iterations increases, and the number of burrows that can be constructed is limited. When $\frac{Max_{iter}}{4}\leq iter<\frac{Max_{iter}}{2}$, the burrow position is updated according to Equation:

(20) $${PD}_{i+1,j+1}={GBest}_{i,j}\;\times \;rPD\;\times \;DS\;\times \;L\overset{’}{e}vy\left(n\right)$$

where

DS: excavation intensity of the coterie.

(21) $${DS=1.5\times r\times \left(1-\frac{iter}{Max_{iter}}\right)}^{\left(2\frac{iter}{Max_{iter}}\right)}$$

where

r: a parameter that is −1 when the iteration number iter is even, and 1 when iter is odd.

iter: current iteration number.

$Max_{iter}$: maximum number of iterations.

#### 4.3.3. Exploitation Process

**Figure 8:**
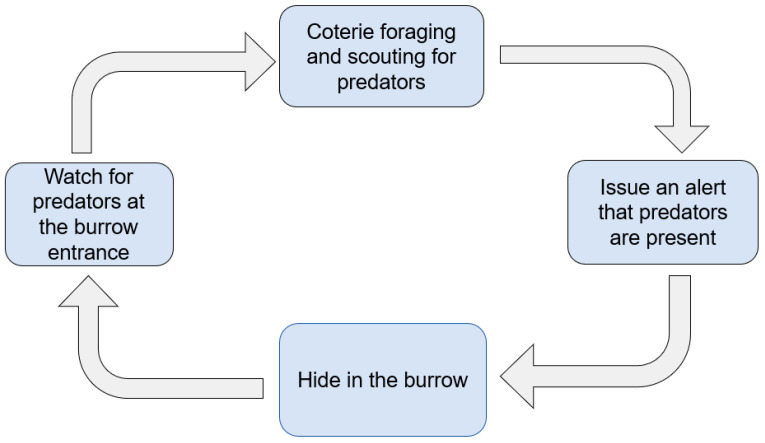
Exploitation phase of the prairie dog optimization algorithm.

The exploitation process of the prairie dog optimization algorithm is shown in Figure 8. In the prairie dog optimization algorithm, prairie dogs communicate using acoustic signals when exploiting the habitat. If a signal indicates a high-quality food source, prairie dogs converge toward its origin. If the signal warns of a predator (e.g., a hawk), prairie dogs in the predator’s path take cover, while others remain at the burrow entrance to monitor the situation [27].

These behaviors cause the population to converge toward regions of higher fitness, enabling a more focused search for optimal or near-optimal solutions. During this exploitation phase, the algorithm concentrates its search within the promising areas identified earlier.

When $\frac{Max_{iter}}{2}\leq iter<3\frac{Max_{iter}}{4}$, the third strategy is applied:

(22) $${PD}_{i+1,j+1}={GBest}_{i,j}\;-{\;eCBest}_{i,j}\;\times \;\varepsilon \;-\;{CPD}_{i,j}\;\times \;rand$$

where

ε: a small parameter representing food source quality (set to 2 × 10^−16^).

rand: a uniformly distributed random number between 0 and 1.

When $3\frac{Max_{iter}}{4}\leq iter\leq Max_{iter}$, the fourth strategy is applied:

(23) $${PD}_{i+1,j+1}={GBest}_{i,j}\;\times \;PE\;\times \;rand$$

where

PE: predator effect.

(24) $${PE=1.5\times \left(1-\frac{iter}{Max_{iter}}\right)}^{\left(2\frac{iter}{Max_{iter}}\right)}$$

The prairie dog optimization algorithm first generates a set of uniformly distributed candidate solutions. It then iteratively explores regions near the current best solution. In each iteration, the algorithm identifies the best solution found so far and updates the incumbent solution accordingly. Swarm intelligence optimization algorithms typically use stopping criteria such as function tolerance, maximum execution time, or a maximum number of iterations [28]. The prairie dog optimization algorithm employs the maximum number of iterations as its stopping criterion.

### 4.4. Prairie Dog Optimization-Based Node Scheduling for Cooperative Tracking

#### 4.4.1. Binary Conversion Function

A transfer function is employed to map the continuous solution to a discrete binary space. Common transfer functions are categorized as S-shaped or V-shaped based on their graphical representation. In this work, the V-shaped transfer function is adopted:

(25) $$T\left(P_{i}\right)=\left|\frac{2}{\pi }arctan\left(\frac{\pi }{2}P_{i}\right)\right|$$

where

$T\left(P_{i}\right)$: the output of the V-shaped transfer function, bounded between 0 and 1.

$P_{i}$: a component of the continuous-valued solution vector generated by the PDO algorithm.

**Figure 9:**
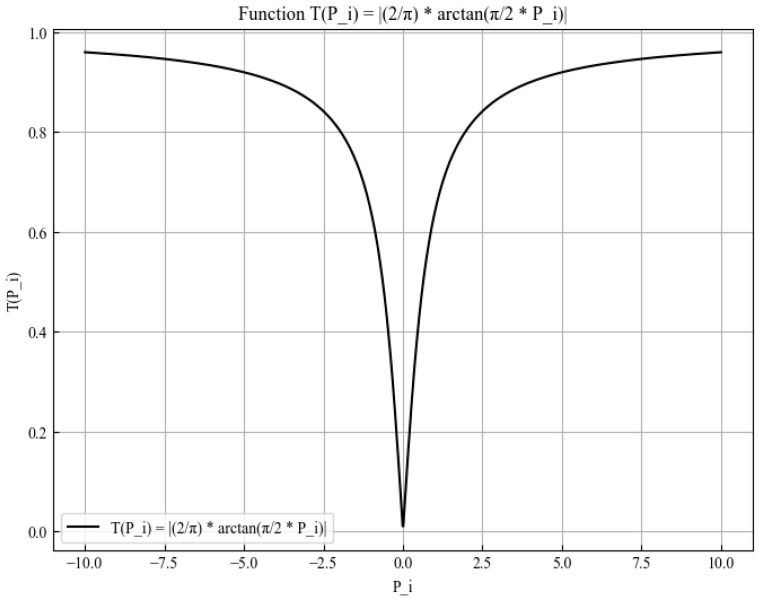
V-shaped binary transfer function.

The V-shaped binary transfer function is shown in Figure 9.

Next, the V-shaped transfer function is used to convert the components of the original solution output by the prairie dog optimization algorithm into binary form:

(26) $$T_{bin}(P_{i})=\{\begin{array}{cc} 1, & T\left(P_{i}\right)>rand,\\ 0, & others. \end{array}$$

where

$T_{bin}(P_{i})$: the final binary value (0 or 1) for the solution component.

rand: a random number uniformly distributed between 0 and 1.

#### 4.4.2. Cauchy Mutation

Cauchy mutation generates new candidate solutions by sampling from the Cauchy distribution, a continuous probability distribution whose probability density function is inversely proportional to the square of the variable. This distribution is characterized by heavy tails, a low peak, and a smooth decay from the peak to zero, which enables it to produce solutions that are distant from the current position.

The probability density function of the standard Cauchy distribution is given by

(27) $$f\left(x;0,1\right)=\frac{1}{\pi \left(1+{\;x}^{2}\right)}$$

where

$f\left(x;0,1\right)$: probability density of value x in a standard Cauchy distribution.

**Figure 10:**
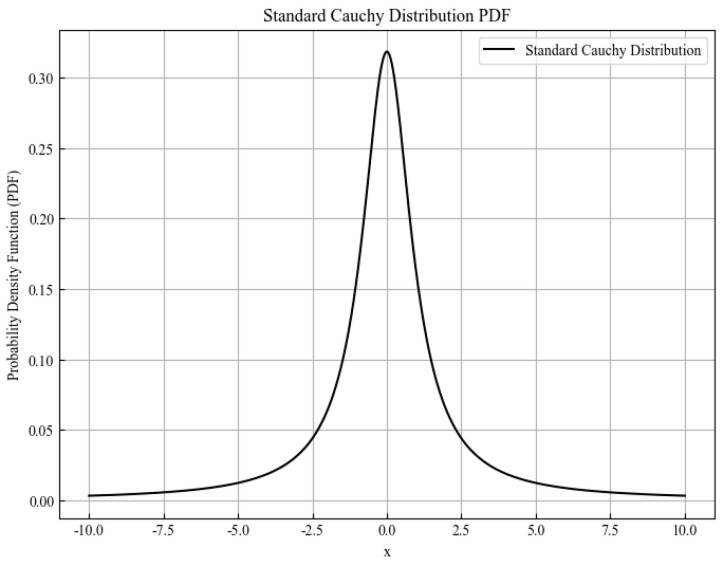
Probability density function of the standard Cauchy distribution.

The probability density function of the standard Cauchy distribution is shown in Figure 10.

In population-based optimization algorithms, Cauchy mutation can be employed to update individual positions. Specifically, the position update is performed as follows:

(28) $$x_{newbest}=x_{best}\;+\;x_{best}\;\times \;\mathrm{cauchy}(0,1)$$

where

$x_{newbest}$: the newly generated candidate solution based on the current best.

$x_{best\;}$: the current best solution.

cauchy(0,1): a random variate sampled from the standard Cauchy distribution.

This update mechanism enhances population diversity, enabling prairie dog individuals to escape local optima and search for the global optimum, thereby aiding the algorithm in exploring a broader region of the problem space.

#### 4.4.3. Hybrid Opposition-Based Learning

The best and worst individuals are two special agents in population-based evolution. The best individual guides the search direction of other members within the coterie, while the worst individual helps maintain population diversity. To mitigate premature convergence and avoid local optima, applying opposition-based learning to the current best solution can increase the probability of escaping local optima and enhance the global exploration capability of the algorithm.

The opposition-based learning operation for the best individual is defined as

(29) $$X_{best}^{\prime }\left(t\right)=lb+\left(u_{b}-X_{best}\left(t\right)\right)$$

where

$X_{best}^{\prime }\left(t\right)$: the opposition-based position of the best individual at iteration *t*.

$X_{best}\left(t\right)$: the original position of the best individual at iteration *t*.

lb and $u_{b}$: the lower and upper bounds of the search space at iteration *t*.

Applying stochastic opposition-based learning to the worst individual in the population can enhance the algorithm’s global exploration capability. The corresponding operation for the worst individual is defined as

(30) $$\;X_{worst}^{\prime }\left(t\right)=lb^{\prime }+rand\times \left(ub^{\prime }-X_{worst}\left(t\right)\right)$$

where

$X_{worst}^{\prime }\left(t\right)$: the opposition-based position of the worst individual.

$X_{worst}\left(t\right)$: the original position of the worst individual.

$lb^{\prime }$ and $ub^{\prime }$: the lower and upper bounds of the search space specifically for the worst individual.

rand: a random value between 0 and 1.

#### 4.4.4. Improved Binary Prairie Dog Optimization for Cooperative Tracking

In cooperative tracking, sensor nodes must be selected according to the operational context. A sensor node typically operates in one of two states: active or sleep. The active state indicates that the node is selected to participate in tracking, whereas the sleep state indicates that it is not selected. The state of the *i*-th sensor can be represented by a binary variable $s_{i}$, where $s_{i}=1$ corresponds to the active state and $s_{i}=0$ corresponds to the sleep state. Consequently, the states of all underwater sensor nodes can be represented as a binary vector, which facilitates the construction of the population and the search for an optimal sensor subset.

When applying the IBPDO algorithm to sensor node selection, the first step is to initialize a population, where each candidate solution corresponds to a prairie dog individual. The set of all individuals forms the candidate population, which is then evaluated using the objective function. The optimization process can be interpreted as the prairie dogs continuously exploring the search space to locate the optimal position.

For the problem considered in this paper, the prairie dog population is represented by an $n\times N_{a}$ matrix, where n is the total number of prairie dog individuals and $N_{a}$ is the total number of candidate underwater sensor nodes.

(31) $$CT=\left[\begin{array}{ccc} s_{1,1} & \cdots & s_{1,N_{a}}\\ \vdots & \ddots & \vdots \\ s_{n,1} & \cdots & s_{n,N_{a}} \end{array}\right]\;s_{i,j}=\left\{\begin{array}{c} 1,select\;the\;sensor\\ 0,do\;not\;select\;the\;sensor \end{array}\right.$$

where

CT: the population matrix representing all candidate sensor selection schemes.

*n*: number of prairie dog individuals (candidate solutions).

$N_{a}$: total number of available sensor nodes in the candidate set.

$s_{i,j}$: a binary variable indicating whether sensor *j* is selected in the *i*-th candidate scheme.

At the beginning of each iteration, the population can be initialized based on the operational context. However, after the population is updated during the algorithm’s iterative process, the number of sensors selected in the new candidate solutions may deviate from $N_{s}$. Therefore, after each iteration, the following repair mechanism is applied to ensure that exactly $N_{s}$ sensors are selected.

If the number of selected sensors ${N_{s}}^{\prime }$ exceeds $N_{s}$, then $N_{s}$ nodes are randomly chosen from the ${N_{s}}^{\prime }$ selected ones. If ${N_{s}}^{\prime }$ is less than $N_{s}$, then $N_{s}\;-{N_{s}}^{\prime }$ nodes are selected with equal probability from the remaining (unselected) sensors.

Algorithm 3 describes the proposed IBPDO algorithm for sensor node selection. The algorithm initializes a population of candidate solutions and iteratively updates their positions through four behavioral phases (foraging, burrowing, food convergence, and predator avoidance) to balance exploration and exploitation. Cauchy mutation and hybrid opposition-based learning are incorporated to enhance global search capability. After each iteration, the V-shaped transfer function converts continuous solutions to binary, and a repair mechanism ensures that exactly $N_{s}$ nodes are selected. The algorithm outputs the globally optimal sensor subset that minimizes the weighted objective function. The specific steps are as described below.
**Algorithm 3.** IBPDO**Input:** The number of prairie dogs *n*, the number of prairie dog family coteries *m*, the attractiveness coefficient *ρ* of the food-source discovery call, the food abundance parameter *ε*, and the required number of sensor nodes to be selected *N_s_*.**Output:** The globally optimal solution found *GBest*.1: Randomly initialize the candidate solutions *CT* and *PD*.2: **while** $iter<{\;Max}_{iter}$ **do**3:    **for** i = 1 to m **do**4:      **for** j = 1 to n **do**5:        Evaluate the fitness of each prairie dog individual.6:        Generate new best and worst candidate solutions using Cauchy mutation and hybrid opposition-based learning, and retain the better of the two.7:        Update the digging strength *DS*, the predator influence, and the randomly accumulated ${CPD}_{i,j}$ of *PE*.8:        **if** $iter<\frac{{Max}_{iter}}{4}$ **then**9:             Simulate the foraging behavior using Equation (15)10:      **else if** $\frac{{Max}_{iter}}{4}\;\leq \;iter<\frac{{Max}_{iter}}{2}$ **then**11:           Simulate the burrowing behavior using Equation (18)12:      **else if** $\frac{{Max}_{iter}}{2}\;\leq iter<3\frac{{Max}_{iter}}{4}$ **then**13:           Simulate the convergence toward food sources using Equation (20)14:      **else**15:           Simulate predator-avoidance behavior using Equation (21)16:      **end if**17:       **end for**18:     **end for**19: Convert the output to binary using the V-shaped transfer function.20: Adjust the number of selected sensor nodes according to *N_s_*21: iter ← iter+122: **end while**23: **return** GBest


After selecting the nodes for the tracking task, the node nearest to the predicted target position is designated as the fusion center. The selected nodes observe the target, employ the UKF to compute local position and covariance estimates, and transmit these to the fusion center. The fusion center then uses Equations (10) and (11) to calculate the fused estimate. Subsequently, the IMM-LSTM-C algorithm proposed in Section 3 is applied to integrate the target motion information over an extended period, thereby obtaining the final target trajectory.

#### 4.4.5. Computational Complexity Analysis

To evaluate the practical feasibility of the proposed IBPDO algorithm for real-time deployment in UASNs, this subsection provides a rigorous theoretical analysis of its computational complexity. Following the standard framework for complexity analysis of swarm intelligence algorithms [29], the computational complexity is primarily determined by three factors: population size, maximum number of iterations, and objective function evaluation.

Let m denote the number of prairie dog family coteries, n the number of prairie dogs per coterie, ${Max}_{iter}$ the maximum number of iterations, and d the dimension of decision variables (i.e., the number of candidate nodes).

Complexity of the original PDO Algorithm: According to the original Prairie Dog Optimization algorithm proposed by Ezugwu et al. [24], its time complexity is given by

(32) $$O\left(PDO\right)=O\left(m\cdot n\cdot {Max}_{iter}\right)\times O(Obj)$$

where O(Obj) represents the complexity of evaluating the objective function (Equation (8)). The objective function involves computing the trace of the posterior covariance matrix and summing the energy consumption of selected nodes. Both operations scale linearly with the number of activated nodes *Ns*, i.e.,$\;O(Obj)=O(N_{s})$.

Additional Overhead Introduced by IBPDO Enhancements: The proposed improvements introduce the following additional computational operations:

Cauchy mutation: Applied only to the current best solution in each iteration, requiring constant time per operation. This adds O(1) per iteration.

Hybrid opposition-based learning: Applied to the best and worst solutions in each iteration, requiring traversal of all dimensions for each operation. This adds O(2d) per iteration.

V-shaped transfer function: Applied to all individuals after each iteration for binary conversion. This adds O(m⋅n⋅d) per iteration.

Node count repair mechanism: Applied to individuals violating the constraint $\sum_{j=1}^{N_{a}}s_{i,j}=N_{s}$ after each iteration. This adds $O(m\cdot n\cdot N_{s})$ per iteration.

Therefore, the overall time complexity of IBPDO is

(33) $$O(IBPDO)=O(m\cdot n\cdot {Max}_{iter}\cdot (d+{\;N}_{s}+Obj))$$

Compared to the original PDO, the increased complexity of IBPDO primarily arises from the linear terms involving dimension d and the number of activated nodes $N_{s}$. Given that in our simulation *d* = 20 and $N_{s}\leq 5$, these additional overheads are practically negligible. More importantly, the complexity of IBPDO is of the same order as other binary swarm intelligence algorithms such as BWOA and BSSA, i.e., $O(pop\cdot {Max}_{\mathrm{iter}}\cdot d)$. This demonstrates that the proposed enhancements improve search capability without significantly increasing computational burden.

### 4.5. Simulation Experiment Analysis

**Figure 11:**
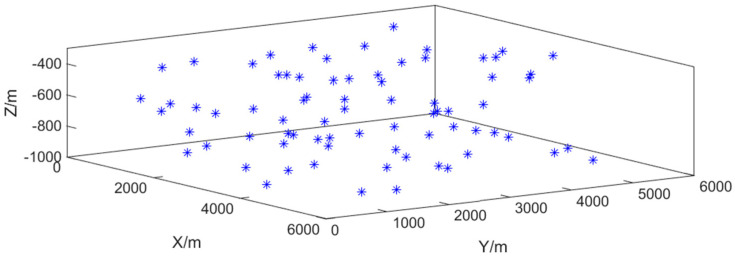
Distribution of sensor positions under the influence of water flow.

The simulation is conducted in a three-dimensional space measuring 6000 m in length, 6000 m in width, and 1000 m in depth. A total of 75 sensor nodes are deployed to observe the moving target. The sensors are distributed across three layers, with 25 sensors per layer. In the top layer, the 25 sensors are arranged in a 5 × 5 square grid, with adjacent sensors spaced 1000 m apart in both the X- and Y-directions. The middle layer is offset by −500 m in the X- and Y-directions relative to the top layer, resulting in a spatial configuration analogous to a body-centered cubic packing. However, due to deployment uncertainties, the horizontal position of each sensor is subject to random errors in both the X- and Y-directions, with a standard deviation of 300 m. Furthermore, because of turbulent underwater currents, the azimuth angle φ between the projection of the anchor chain onto the seafloor and the *X*-axis follows a uniform distribution over [0,2π], and the inclination angle θ between the anchor chain and the vertical direction is uniformly distributed over [0,π/4]. The sensor position distribution affected by water flow is shown in Figure 11.

In the energy consumption model, each underwater acoustic transmission carries a payload of 512 bits. The energy required to transmit or receive one bit is 2 nJ. The minimum received signal power is set to 50 $µJ\cdot {bit}^{-1}\cdot {km}^{-2}$, the energy diffusion factor is 2, and the absorption coefficient is 20 dB/km.

**Figure 12:**
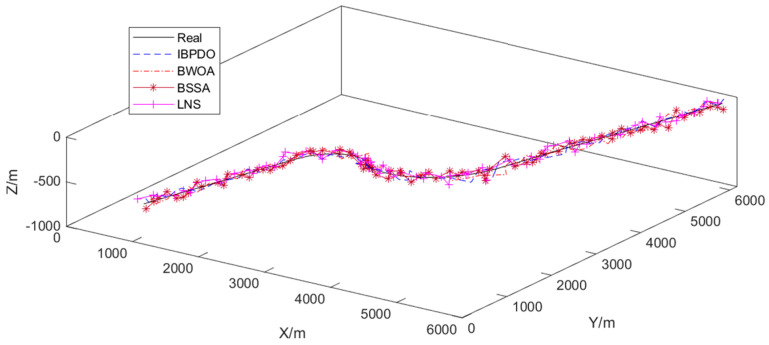
Collaborative tracking using different node selection algorithms.

The proposed IBPDO algorithm is compared with the BWOA, the BSSA, and the LNS algorithm based on local information. Figure 12 presents the collaborative tracking trajectories obtained using different node selection algorithms (IBPDO, BWOA, BSSA, and LNS). The horizontal and vertical axes represent the X and Y coordinates in meters, respectively. The black solid line indicates the true target trajectory, while the colored dashed lines show the estimated trajectories from each algorithm. It can be observed that the trajectory estimated by the proposed IBPDO algorithm aligns most closely with the true trajectory, particularly during the turning maneuvers around (X ≈ 2000 m, Y ≈ 3000 m) and (X ≈ 4000 m, Y ≈ 1500 m). In contrast, the trajectories produced by BWOA, BSSA, and LNS exhibit noticeable deviations from the true path, indicating that IBPDO achieves superior node selection and, consequently, higher tracking accuracy.

The population size is set to 100 individuals, the maximum number of iterations is 1000, the upper bound is 4, and the lower bound is 0. For the IBPDO algorithm, the attractiveness coefficient ρ of the food-source discovery call is set to 0.1, the food-source quality parameter ε is set to $2\times 10^{-16}$, and the individual difference parameter Δ is set to 0.005. In a realistic UASN environment, nodes far from the target suffer from severe signal attenuation, so their observations contribute negligibly to the fusion estimation while introducing excessive communication overhead. Therefore, to reduce the dimensionality and computational complexity of the optimization problem, this study selects only the 20 nearest nodes to the target as the candidate set at each time step. This setup not only simulates the communication range constraint of cluster heads in real-world scenarios but also ensures a fair comparison among all competing algorithms under the same search space. The 20 sensor nodes closest to the target are selected, corresponding to 20 coteries. The target trajectory follows the motion pattern defined in Section 3.2.

**Figure 13:**
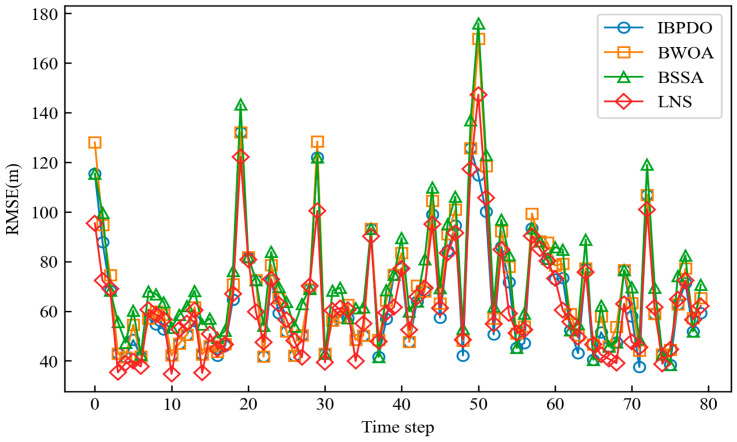
RMSE error of collaborative tracking when using different node selection algorithms.

Figure 13 presents the root mean square error (RMSE) of cooperative tracking achieved by the IBPDO, BWOA, and BSSA algorithms. The horizontal axis represents the time step, and the vertical axis represents the RMSE in meters (m). In each time step, four sensor nodes are selected from the available nodes. The performance of the LNS algorithm is also included for comparison. The results indicate that the IBPDO algorithm achieves a lower final tracking RMSE than the other swarm-intelligence-based node selection algorithms. This demonstrates that the IBPDO possesses strong search capability, enabling it to select a superior subset of sensor nodes and consequently deliver better tracking performance.

**Figure 14:**
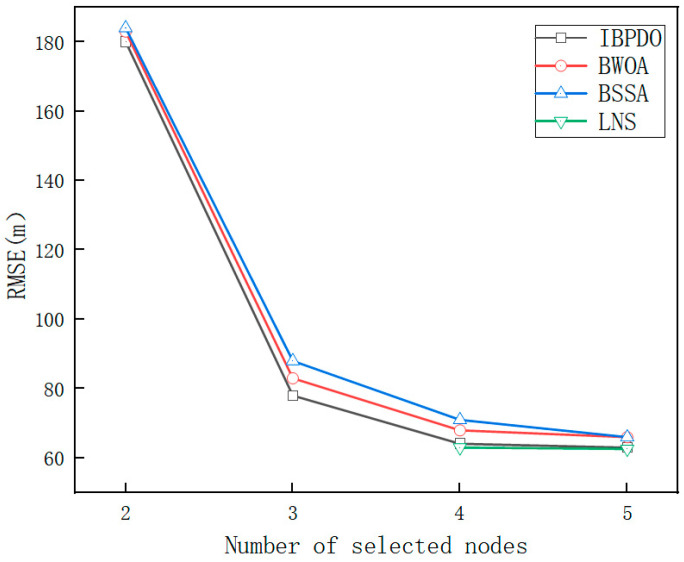
Effect of choosing different numbers of sensor nodes per time step on tracking performance.

Through 100 Monte Carlo experiments, we investigated the influence of the number of sensor nodes selected per time step on tracking performance. As shown in Figure 14, as the number of activated sensor nodes in each time step increases, the cooperative tracking error decreases significantly. This is because selecting and activating more sensors within a time step provides more measurement information, which helps mitigate observation errors after data fusion and ultimately enhances tracking accuracy.

When two nodes are selected, few sensors are activated, so the system suffers from a lack of spatial diversity and measurement redundancy. Regardless of which optimization algorithm is used, the total amount of information collected is insufficient to effectively mitigate observation noise. In this information-scarce regime, the tracking accuracy is constrained by the physical limitation of insufficient data rather than the intelligence of the selection algorithm. Therefore, all algorithms (IBPDO, BWOA, BSSA) exhibit similarly high RMSE. With three nodes selected, the RMSE of the IBPDO algorithm is reduced by 8.2% compared to the BWOA algorithm. When four nodes are selected, the error of the IBPDO algorithm is reduced by 6.1% relative to the BWOA algorithm. When five nodes are selected, the number of selected nodes increases, and the candidate set becomes highly information-redundant. Activating 5 out of the 20 nearest nodes ensures that almost any reasonable subset provides sufficient spatial coverage and measurement diversity. In this information-redundant regime, the marginal benefit of further optimization diminishes, and the performance gap between different algorithms narrows. Consequently, all algorithms achieve similarly low RMSE. In the simulation, the number of nodes selected by the LNS algorithm in one time step is variable, typically around four or five; its RMSE is 63.6 m, which is comparable to the RMSE of 64.1 m achieved by the IBPDO algorithm with four nodes selected.

Although, in theory, greater sensor participation improves cooperative tracking accuracy, the rate of accuracy improvement diminishes and may even stagnate once the number of selected active sensor nodes exceeds a certain threshold. This occurs because an excessive number of sensors leads to information redundancy. Furthermore, increasing the number of sensor nodes raises system complexity and incurs unnecessary energy consumption. Therefore, the number of nodes should be selected appropriately according to the operational requirements.

**Figure 15:**
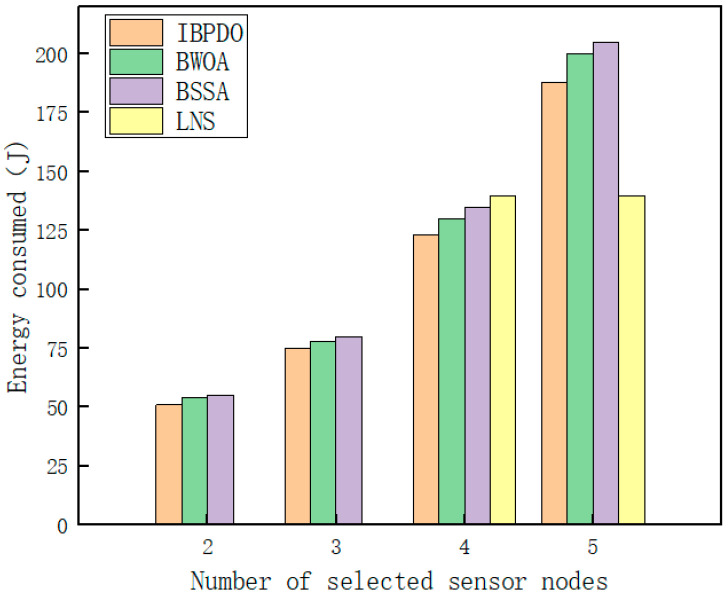
Effect of choosing different numbers of sensor nodes per time step on cooperative tracking performance.

The influence of the number of sensor nodes selected per time step on tracking performance was further analyzed. In the simulation, the number of nodes selected by the LNS algorithm in a single time step varies, typically between four and five, with an associated energy consumption of 139.8 J. As shown in Figure 15, although the cooperative tracking error decreases continuously as more underwater acoustic sensor nodes are activated, the energy consumption of the underwater acoustic sensor network also rises. When three sensors are selected, the network energy consumption using the IBPDO algorithm for node selection is 4.8% lower than that of the BWOA algorithm and 7.5% lower than that of the BSSA algorithm. When four nodes are selected, and the tracking accuracy is comparable to that of the LNS algorithm, and the energy consumption of the IBPDO algorithm is reduced by 12% relative to the LNS algorithm, by 5.3% relative to the BWOA algorithm, and by 6.9% relative to the BSSA algorithm.

It can also be observed that selecting three to four sensors per time step achieves a favorable balance between tracking accuracy and energy consumption. Node selection algorithms such as LNS may activate an excessive number of sensor nodes, leading to unnecessary energy expenditure in underwater communication. In contrast, the IBPDO algorithm can select a more energy-efficient sensor subset, thereby avoiding the activation of redundant nodes and maintaining tracking accuracy with lower energy consumption.

To further assess the practical feasibility of the proposed IBPDO algorithm for real-world deployment, this subsection presents an experimental evaluation of its computational efficiency in comparison with BWOA, BSSA, and LNS. Table 3 reports the average computation time required for node selection at each time step under different numbers of selected nodes.

Table 3 presents the average execution time per time step for different node selection algorithms. Among the four algorithms, IBPDO achieves the lowest computational overhead among all swarm intelligence algorithms, with execution times ranging from 0.10 to 0.20 ms per time step—approximately 65–80% lower than BWOA (0.53–0.56 ms) and 47–71% lower than BSSA (0.38–0.45 ms). LNS, as a heuristic method, shows relatively low computation time (0.13–0.30 ms) but exhibits higher variability as node count increases. IBPDO also demonstrates excellent scalability, with only a 0.10-millisecond increase when node count grows from 2 to 5. Considering the underwater acoustic propagation delay (typically 4–10 s) and the 10-s sampling interval in our simulation, the computational overhead of IBPDO is negligible for practical deployment. These results confirm that IBPDO not only achieves superior accuracy–energy trade-offs (as demonstrated in Figure 12, Figure 13 and Figure 14) but also offers the best computational efficiency among the compared swarm intelligence algorithms, making it highly suitable for resource-constrained UASN applications.

**Figure 16:**
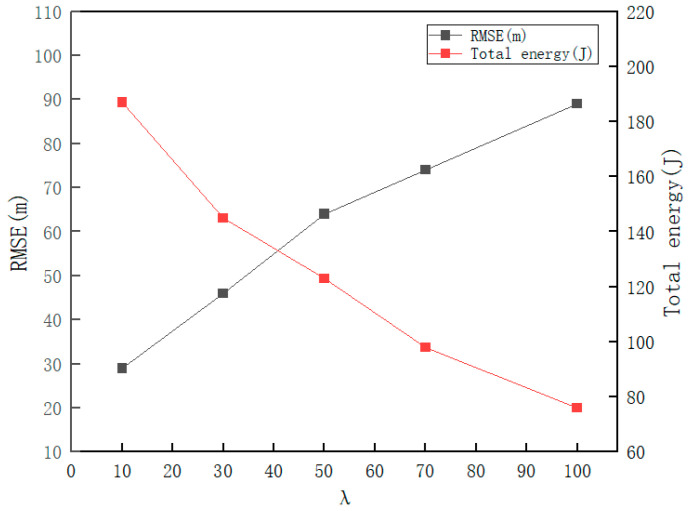
Sensitivity analysis of the weight coefficient λ on tracking RMSE and total energy consumption.

Figure 16 presents a sensitivity analysis of the weight coefficient λ on the average tracking RMSE and total energy consumption of the proposed IBPDO node scheduling scheme. As indicated by Equation (8), λ serves as the weight for the total node energy consumption: the larger the value of λ, the lower the objective function is intended to be, and the most effective approach to achieve this is to reduce the total energy consumption. Through experiments, we obtained a visual trade-off curve illustrating the relationship between accuracy and energy consumption of the IBPDO scheme with varying λ values (ranging from 0 to 100): the horizontal axis represents λ (0~100), the left vertical axis denotes RMSE (m), and the right vertical axis represents total energy consumption (J). As can be observed from the figure, with the increase of λ, the RMSE increases monotonically (rising from approximately 29.5 m to about 89 m), while the total energy consumption decreases monotonically (dropping from around 189 J to roughly 76 J). This demonstrates that a larger λ makes the algorithm more inclined toward energy conservation, with fewer nodes selected for participation, and the tracking error increases accordingly. At λ = 50, the RMSE is approximately 64.5 m and the energy consumption is about 123 J, which falls within the trade-off range between tracking accuracy and energy consumption. Therefore, λ is a key parameter for adjusting the balance between “tracking accuracy” and “energy consumption”. In practical deployment, an appropriate λ value can be selected from the figure according to the requirements for tracking accuracy and network lifetime.

As shown in Table 4, the proposed complete joint framework (Scheme 5) achieves the optimal balance in both tracking accuracy and energy consumption. In terms of tracking accuracy, Scheme 5 achieves an RMSE of 64.1 m, which represents a reduction of 19.3% compared to the traditional IMM+LNS scheme (Scheme 1), 11.9% compared to Scheme 2 using only IBPDO node selection, 9.1% compared to Scheme 3 using only IMM-LSTM-C tracking, and 3.3% compared to Scheme 4 using BWOA node selection. These results demonstrate a significant synergistic effect between IMM-LSTM-C and IBPDO: IBPDO selects sensor subsets with higher information quality for the tracking algorithm, while IMM-LSTM-C more effectively utilizes these high-quality observations for accurate state estimation.

In terms of network energy consumption, Scheme 5 achieves a total energy consumption of 123.0 J, which is significantly lower than Scheme 1 (a reduction of 15.4%) and Scheme 3 (a reduction of 12.8%), while remaining comparable to Scheme 2 (124.5 J) and outperforming Scheme 4 (130.5 J) by 5.7%. Notably, Scheme 5 achieves higher tracking accuracy (64.1 m vs. 72.8 m) with energy consumption comparable to Scheme 2, demonstrating that the IBPDO node selection strategy, when integrated with IMM-LSTM-C, can more effectively identify and activate energy-efficient sensor nodes, achieving the optimization objective of “higher accuracy at equivalent energy consumption.”

In summary, the experimental results demonstrate that the proposed IMM-LSTM-C + IBPDO joint framework is not a simple superposition of two algorithms but achieves synergistic optimization of tracking accuracy and energy consumption. By dynamically scheduling sensor resources according to the target’s motion state, the framework maintains high-precision tracking while effectively extending network lifetime, fully validating the necessity and superiority of the joint optimization design.

## 5. Conclusions

This paper delves into the target tracking problem in underwater acoustic sensor networks and proposes a high-precision, low-energy collaborative tracking scheme. First, to address the tracking accuracy issue, the IMM-LSTM-C algorithm based on LSTM motion model classification is proposed. By fusing the likelihood function values of the interactive multi-model over multiple time steps, this algorithm effectively enhances the recognition accuracy of motion patterns, thereby improving the target tracking accuracy by approximately 7.1%. Second, to tackle the network energy consumption problem, the IBPDO node selection and scheduling algorithm based on improved binary prairie dog optimization is introduced. This algorithm enhances global search capability by incorporating Cauchy mutation and hybrid opposition-based learning strategies, and it balances estimation accuracy and network energy consumption within the objective function. Simulation results demonstrate that the IBPDO algorithm reduces network energy consumption by 12% while maintaining tracking accuracy comparable to traditional algorithms.

Further theoretical complexity analysis and experimental runtime comparisons reveal that the computational complexity of the IBPDO algorithm is comparable to mainstream swarm intelligence algorithms such as BWOA and BSSA, and its single-step execution time is significantly lower than the underwater acoustic propagation delay. These results fully validate the computational feasibility of the IBPDO algorithm in practical deployment scenarios for UASNs.

A limitation of this study is the absence of explicit modeling of Doppler shift in the underwater acoustic (UWA) channel, although the current framework assumes that physical layer compensation is handled by the underlying communication system. Future work will explore cross-layer joint optimization methods that incorporate channel state information, including Doppler spread, into the node selection criteria to better adapt to the actual transmission characteristics of the underwater acoustic channel.

## Figures and Tables

**Table 1:** Simulation parameters.

Parameter	Value
Monitoring area	6000 m × 6000 m × 1000 m
Total number of nodes	75
Node deployment	3 layers, 5 × 5 grid, 500 m offset
Packet size	512 bits
Tx/Rx energy per bit	2 nJ/bit
Absorption coefficient	20 dB/km
Balancing coefficient λ	50
Optimizer	AdamW
Weight decay	1 × 10^−4^
Scheduler	Cosine Annealing

**Table 2:** Root mean square error for different tracking algorithms.

Tracking Algorithm	RMSE
IMM	72.19
IMM-BP	69.01
IMM-LSTM-C	64.11

**Table 3:** Average execution time per time step (ms).

Algorithm	2 Nodes	3 Nodes	4 Nodes	5 Nodes
IBPDO	0.10	0.13	0.18	0.20
BWOA	0.56	0.54	0.56	0.53
BSSA	0.38	0.45	0.44	0.39
LNS	0.13	0.19	0.30	0.26

**Table 4:** Overall Performance Comparison of Different Tracking and Node Selection Combinations.

Scheme	Tracking Algorithm	Node Selection Algorithm	RMSE (m)	Total Energy Consumption (J)
Scheme 1	IMM	LNS	79.4	145.3
Scheme 2	IMM	IBPDO	72.8	124.5
Scheme 3	IMM-LSTM-C	LNS	70.5	141.0
Scheme 4	IMM-LSTM-C	BWOA	66.3	130.5
Scheme 5	IMM-LSTM-C	IBPDO	64.1	123.0

## Data Availability

Data is contained within the article.
